# Home-Based Gamma Transcranial Alternating Current Stimulation in Patients With Alzheimer Disease: A Randomized Clinical Trial

**DOI:** 10.1001/jamanetworkopen.2025.46556

**Published:** 2025-12-08

**Authors:** Valentina Cantoni, Elias Paolo Casula, Barbara Tarantino, Chiara Cupidi, Nadine Huber, Daniele Altomare, Enrico Premi, Elisa Zummo, Romina Esposito, Carla Leonardi, Sanna-Kaisa Herukka, Eino Solje, Asia Ferrari, Maria Sofia Cotelli, Roberto Gasparotti, Alessandro Martorana, Claudia Fracassi, Emiliano Santarnecchi, Giacomo Koch, Annakaisa Haapasalo, Mario Grassi, Alberto Benussi, Barbara Borroni

**Affiliations:** 1 Department of Continuity of Care and Frailty, Azienda Socio Sanitaria Territoriale (ASST) Spedali Civili of Brescia, Brescia, Italy; 2 Department of Clinical and Experimental Sciences, University of Brescia, Brescia, Italy; 3 Department of Clinical and Behavioural Neurology, Santa Lucia Foundation Istituto di Ricovero e Cura a Carattere Scientifico (IRCCS), Rome, Italy; 4 Department of System Medicine, Tor Vergata University, Rome, Italy; 5 Department of Brain and Behavioural Sciences, Medical and Genomic Statistics Unit, University of Pavia, Pavia, Italy; 6 Neurology Unit, Fondazione Istituto G. Giglio, Cefalù, Italy; 7 A. I. Virtanen Institute for Molecular Sciences, University of Eastern Finland, Kuopio; 8 Stroke Unit, ASST Spedali Civili, Brescia, Italy; 9 Institute of Clinical Medicine, University of Eastern Finland, Kuopio; 10 Neuro Center, Neurology, Kuopio University Hospital, Kuopio, Finland; 11 Neuroradiology Unit, Department of Medical and Surgical Specialties, University of Brescia, Brescia, Italy; 12 Department of Systems Medicine, Memory Clinic, University of Rome Tor Vergata, Rome, Italy; 13 Neurophysiology Lab, IRCCS Istituto Centro San Giovanni di Dio Fatebenefratelli, Brescia, Italy; 14 Precision Neuroscience and Neuromodulation Program, Gordon Center for Medical Imaging, Department of Radiology, Massachusetts General Hospital, Harvard Medical School, Boston; 15 Department of Neuroscience and Rehabilitation, University of Ferrara, Ferrara, Italy; 16 Center for Translational Neurophysiology of Speech and Communication, Italian Institute of Technology, Ferrara, Italy; 17 Neurology Unit, Department of Medical, Surgical and Health Sciences, University of Trieste, Trieste, Italy; 18 Molecular Markers Laboratory, IRCCS Istituto Centro San Giovanni di Dio Fatebenefratelli, Brescia, Italy

## Abstract

**Importance:**

Alzheimer disease (AD) is characterized by dysregulated gamma brain oscillations. Transcranial alternating current stimulation (tACS) is a novel, noninvasive brain stimulation technique capable of entraining cerebral oscillations at targeted frequencies.

**Objective:**

To assess the safety, feasibility, and efficacy of home-based gamma tACS applied over the precuneus in patients with prodromal and mild AD.

**Design, Setting, and Participants:**

This double-blind, randomized, sham-controlled clinical trial with an open-label extension phase was conducted at a tertiary AD research clinic in Italy from December 10, 2022, to October 15, 2024. Patients with a diagnosis of AD were eligible to participate.

**Intervention:**

Participants were randomized to receive either home-based gamma tACS (5 sessions/wk, 60 minutes each) or sham stimulation for 8 weeks (double-blind phase). All participants subsequently received gamma tACS for an additional 8 weeks (open-label phase) and an 8-week follow-up.

**Main Outcomes and Measures:**

The primary end points were safety, feasibility, and clinical efficacy. Secondary end points included measures of biological efficacy, including gamma band power via electroencephalography, cholinergic neurotransmission, AD plasma biomarker levels, and brain connectivity as assessed via magnetic resonance imaging.

**Results:**

Sixty consecutive patients with prodromal or mild AD were screened; 50 were randomized to gamma or sham tACS (mean [SD] age, 67.3 [7.8] years; 25 [50.0%] female and 25 [50.0%] male). Home-based gamma tACS was safe and well-tolerated. A significant enhancement in global cognitive functions, activities of daily living, and associative memory performances was observed. Marginal mean differences between the sham vs gamma tACS groups were significant for the Clinical Dementia Rating sum of boxes (0.35; 95% CI, 0.10-0.61; *P* = .007), Alzheimer Disease Assessment Scale–cognitive subscale (0.93; 95% CI, 0.50-1.36; *P* = .001), Alzheimer Disease Cooperative Study–Activities of Daily Living (−0.55; 95% CI, −0.89 to −0.21; *P* = .02), and Face-Name Association Test (−1.14; 95% CI, −1.66 to −0.61; *P* ≤ .001). During the open-label phase, a significant marginal mean difference was observed for Alzheimer Disease Assessment Scale–cognitive subscale (−0.59; 95% CI, −1.02 to −0.16; *P* = .007), Alzheimer Disease Cooperative Study–Activities of Daily Living (0.41; 95% CI, 0.04-0.08; *P* = .02), and Face-Name Association Test (1.04; 95% CI, 0.50-1.57; *P* = .003). Neurophysiological measures showed an increase in cholinergic transmission, coinciding with an increase in gamma power following gamma tACS, effects not seen with sham stimulation. No changes of plasma biomarkers were observed. No add-on effect was observed after 2 repeated treatments with gamma tACS, suggesting that 8 rather than 16 weeks of treatment represents the ideal duration.

**Conclusions and Relevance:**

In this randomized clinical trial, home-based gamma tACS was feasible and improved clinical outcomes in AD, with neurophysiological evidence of brain engagement. These findings support further investigation of gamma tACS as a potential therapeutic intervention for AD.

**Trial Registration:**

ClinicalTrials.gov Identifier: NCT05643326

## Key Points

**Question:**

Is gamma–transcranial alternating current stimulation (tACS) over the precuneus effective in a home-based setting for patients with Alzheimer disease?

**Findings:**

In this randomized clinical trial including 50 patients with prodromal or mild Alzheimer disease, gamma tACS was found to be safe, well tolerated, and clinically effective, with improvements observed in indirect measures of cholinergic neurotransmission. Additionally, gamma tACS demonstrated the ability to entrain gamma rhythms over the parietal and frontal regions.

**Meaning:**

Gamma tACS represents a promising therapeutic approach for Alzheimer disease, offering potential insights into novel treatment strategies.

## Teaser

This randomized clinical trial uses a double-blind sham-controlled phase followed by an open-label phase to establish proof of concept for home-based gamma transcranial alternating current stimulation in patients with mild Alzheimer disease.

## Introduction

Alzheimer disease (AD), the leading cause of dementia worldwide, is primarily characterized by amyloid-β and tau protein accumulations in the brain.^1^ Despite the advent of cholinergic enhancement as a cornerstone of AD therapeutics, the recent approval of anti–amyloid monoclonal antibodies has introduced a new era of potential disease-modifying treatments.^2^ However, given the multifaceted and complex pathophysiological mechanisms underlying AD, there remains a critical need for additional therapeutic approaches to advance clinical management.

Growing evidence highlights that AD is associated with a pronounced disruption of physiological gamma oscillations (30-80 Hz) in the brain, which are integral for cognitive processes such as attention, memory, and perception.^3^ Hence, restoring gamma oscillatory activity through neuronal entrainment is a compelling therapeutic intervention in AD. Preclinical studies in animal models have demonstrated that sensory stimulation, such as flickering light and sound at gamma frequencies, induces gamma oscillations across multiple brain areas, reduces amyloid deposition, and improves cognitive outcomes.^4,5,6^ An early-stage human trial^7^ has extended these findings, showing that gamma-frequency sensory stimulation is safe and potentially effective in improving cognitive and functional measures in patients with mild AD.

However, age-related sensory impairments (eg, visual or auditory deficits) and the limited reach of sensory-driven neural activity into brain networks most affected by AD pathology may constrain the clinical efficacy of these approaches. To address these limitations, we used transcranial alternating current stimulation (tACS), a noninvasive neuromodulation technique that directly targets brain oscillations. tACS delivers sinusoidal alternating electrical currents via the scalp, entraining neuronal firing patterns to the desired frequency.^8^

Prior pilot studies that included several of the present investigators^9,10,11^ demonstrated that a single session of gamma tACS over the precuneus, a key hub of the default mode network and one of the earliest regions affected in AD, was safe and effective in enhancing cholinergic neurotransmission. Using transcranial magnetic stimulation (TMS) paired-pulse protocols, Benussi et al^10,11^ observed restoration of short-latency afferent inhibition (SAI), an indirect marker of cholinergic activity. However, the cumulative and long-lasting effects of repeated gamma tACS sessions remain unknown. Additionally, the impact of gamma tACS on AD-related plasma biomarkers has yet to be clarified.

We conducted a double-blind, randomized, sham-controlled clinical trial followed by an open-label phase to establish proof of concept for home-based gamma tACS in patients with mild AD. Specifically, we aimed to: (1) assess the safety and feasibility of at-home gamma tACS delivery; (2) evaluate the short- and long-term effects of multisession gamma tACS on cognitive functions, cholinergic transmission, and blood biomarkers; (3) determine the persistence of treatment effects over time to optimize the timing for repeated interventions; and (4) explore the modulation of brain entrainment and connectivity.

## Methods

This randomized clinical trial was approved by the local ethics committee of Brescia Hospital, Brescia, Italy. Written informed consent was obtained from all participants in accordance with the Declaration of Helsinki. The study followed the Consolidated Standards of Reporting Trials (CONSORT) reporting guideline. The study protocol is found in Supplement 1.

### Participants

Participants fulfilling current clinical criteria for AD^12^ were screened and included in the studyat the Neurology Unit, Department of Clinical and Experimental Sciences, University of Brescia, Brescia, Italy (eFigure 1 in Supplement 2). Each participant underwent standardized neuropsychological testing, routine blood analysis, structural imaging, and either positron emission tomographic amyloid imaging or cerebrospinal fluid analysis to measure amyloid-β_1-42_, tau, and phosphorylated tau_181_ levels. Only patients in their prodromal or mild stage (Clinical Dementia Rating Scale score, 0.5 or 1.0) and with positive positron emission tomographic amyloid and/or cerebrospinal fluid AD-related pattern were considered (inclusion and exclusion criteria are reported in the eMethods in Supplement 2). Caregivers’ demographic data were recorded, and cognitive screening (Mini-Mental State Examination score >27 of 30 points possible) was performed to ensure unimpaired cognition.

### Study Design

**Figure 1.**
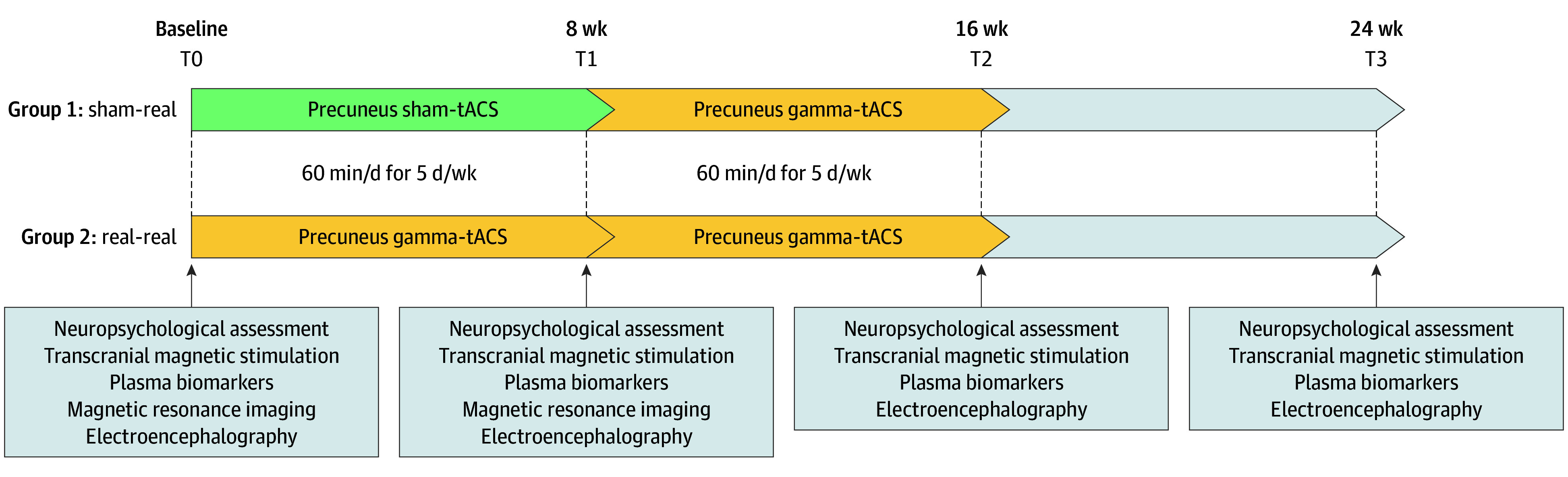
Study Design tACS indicates transcranial alternating current stimulation.

Patients were randomized into 2 groups in a 1:1 ratio for the initial controlled phase. At baseline (T0), group 1 received sham tACS, while group 2 received gamma tACS for 5 d/wk for 8 weeks (T1; randomized, double-blind, sham-controlled phase). All participants then received gamma tACS for an additional 8 weeks (T2; open-label phase) and an 8-week follow-up (T3) (Figure 1).

At each time point (T0-T3), every patient underwent a cognitive and behavioral evaluation according to a standardized protocol, TMS measurements indirectly assessing cholinergic circuits, blood sampling for biomarker evaluation, and electroencephalographic (EEG) recordings for brain entrainment analysis. Moreover, at baseline (T0) and 8-week follow-up (T1), a subgroup of patients underwent magnetic resonance imaging (MRI) of the brain for connectivity assessment. At the end of the study, caregivers’ perceived burden during the stimulation period was evaluated using a Likert scale in which 0 indicated no burden and 10 indicated high burden.

The participants and the examiners performing clinical ratings, tACS, TMS, EEG, and MRI protocols were blinded to the type of stimulation. A single investigator (B.B.) was responsible for random allocation sequences, enrollment of participants, allocation concealment, and assignment of participants to specific interventions.

### Outcome Measures

The primary end points were defined as (1) safety and feasibility and (2) clinical efficacy. The secondary end points were defined as changes of cholinergic neurotransmission measurements, plasma biomarkers, functional brain connectivity using MRI, and gamma brain entrainment using EEG.

### Cognitive Assessment

Cognitive and behavioral assessment included the Clinical Dementia Rating sum of boxes (CDR-SB),^13^ the Alzheimer Disease Assessment Scale–cognitive subscale, 13-item version (ADAS-Cog-13),^14^ the Alzheimer Disease Cooperative Study–Activities of Daily Living (ADCS-ADL),^15^ the Face-Name Association Test (FNAT),^16^ the Rey Auditory Verbal Learning Test,^17^ the semantic fluency task, the Trail Making Test,^18^ and the Neuropsychiatric Inventory.^19^ Caregiver burden was assessed with the Caregiver Burden Inventory^20^ (eMethods in Supplement 2).

### TMS Assessment

A TMS figure-of-eight coil was used to assess short latency afferent inhibition (SAI) (an indirect marker of cholinergic transmission), with a paired-pulse technique using a conditioning-test design, as previously reported^11,21^ (further details are provided in the eMethods in Supplement 2).

### Plasma Biomarkers Assessment

Plasma samples were collected according to standard procedures and stored at −80°C until use. Plasma levels of neurofilament light , glial fibrillary acidic protein, amyloid β_1-42_, and amyloid β_1-40_ were quantified using a commercial assay kit (Neurology 4-plex E 103670; Quanterix), and plasma levels of phosphorylated tau_217_ was quantified using a separate assay (ALZpath; Quanterix) according to manufacturer instructions. Samples were run in duplicate on an automated bead-based immunoassay platform (Simoa-HD-X; Quanterix).

### MRI Preprocessing and Analysis

A subgroup of patients with AD underwent MRI scanning (Skyra 3T; Siemens), including structural T1-weighted magnetization prepared rapid gradient echo images and 200 functional blood oxygenation level–dependent volumes. After undergoing standard preprocessing using the FMRIB Software Library,^22^ a seed-based analysis was used to examine the precuneus region’s connectivity.^23^ The generated maps were used to model within-participant and between-group differences (T0-T1) with a 2-way analysis of variance (further details are provided in the eMethods in Supplement 2).

### EEG Recordings and Analysis

EEG data were recorded using a slim 64-channel active electrode system (actiCAP; Brain Products GmbH) connected to an amplifier (actiCHamp Plus 64 System; Brain Products GmbH). Data were preprocessed in MATLAB software, release 2024a (The Mathworks) using the EEGLAB toolbox (University of California, San Diego)^24^ and custom scripts (further details on EEG preprocessing and analysis are provided in the eMethods in Supplement 2).

### tACS Administration and Monitoring

tACS was delivered using a mini–computed tomographic device (Soterix Medical), through a pair of saline-soaked (0.9% sodium chloride) surface sponge electrodes placed on the scalp over the precuneus (centered over the midline parietal site, Pz, according to the 10-20 international EEG coordinates) and over the right deltoid muscle. A custom headset was personalized for each participant. The electrodes were secured using elastic gauzes, and electroconductive gel was applied to electrodes to reduce contact impedance (<5 kΩ for all sessions). During tACS stimulation, a sinusoidal current of 3 mA peak to trough (1.5 mA peak to baseline) was applied for 60 minutes, while during sham stimulation, the current was discontinued 60 seconds after initiation to mimic the sensation of real stimulation. As previously reported, ^25^ participants and caregivers received in-person training during the first week, regardless of randomization. In subsequent weeks, tACS sessions were performed at participants’ homes with caregiver involvement. Remote monitoring by the study team ensured adherence, including verification of participant-entered session codes before each stimulation and oversight via video calls. All participants were provided with the stimulation device free of charge for the entire duration of the trial, ensuring equal access and minimizing potential selection bias related to socioeconomic status or educational level.

### Statistical Analysis

To assess the effect of tACS treatment on clinical, biological, and neurophysiological measures over time, we used a mixed model (eMethods in Supplement 2).^26,27^ Fixed-effect coefficients and their 95% CIs were reported using marginal mean differences when the interaction terms were significant, or conditional mean differences for main effects. EEG data were analyzed with a 2-way analysis of variance with time and treatment as fixed factors (eMethods in Supplement 2). Statistical analyses were performed with R, version 4.2.2 (R Project for Statistical Computing). Two-sided *P* < .05 indicated statistical significance.

## Results

### Participants

Fifty-three participants were initially enrolled in the study, with 3 of them dropping out (eFigure 1 in Supplement 2). Thus, 50 participants (mean [SD] age, 67.3 [7.8] years; 25 female [50.0%] and 25 male [50.0%]) were included in the final analysis. Demographic and clinical characteristics are presented in Table 1. Caregiver demographics, including age, sex, and relationship to the participant, were comparable across groups.

**Table 1.** Baseline Characteristics of Patients and Caregivers^a^

Characteristic	All patients (N = 50)	Group 1 (n = 24)	Group 2 (n = 26)
**Patients**			
Age, y	67.3 (7.8)	68.3 (8.5)	66.4 (7.1)
Sex, No. (%)			
Female	25 (50.0)	12 (50.0)	13 (50.0)
Male	25 (50.0)	12 (50.0)	13 (50.0)
Age at onset of AD, y	64.6 (7.8)	65.8 (8.2)	63.5 (7.3)
Family history of AD, positive, No. (%)	16 (32.0)	8 (33.3)	8 (30.8)
Educational attainment, y	12.2 (4.0)	11.5 (4.0)	12.8 (4.0)
Clinical assessments at baseline			
MMSE score^b^	23.9 (2.4)	23.9 (2.6)	23.9 (2.5)
CDR-SB score^c^	2.5 (1.4)	2.7 (1.6)	2.3 (1.2)
ADAS-Cog-13 score^d^	17.8 (5.4)	17.8 (4.6)	17.9 (5.4)
ADCS-ADL score^e^	66.2 (7.9)	65.2 (8.9)	67.2 (6.7)
FNAT score^f^	7.2 (2.4)	7.4 (2.6)	7.1 (2.2)
RAVL score^g^			
Immediate recall	21.6 (9.3)	20.4 (8.5)	22.6 (10.1)
Delayed recall	1.7 (2.1)	1.5 (2.1)	2.0 (2.1)
Semantic fluency score^h^	14.8 (4.9)	14.8 (5.1)	14.7 (4.9)
TMT score			
Test A^i^	74.4 (63.6)	81.3 (70.3)	68.0 (57.4)
Test B^j^	341.0 (187.4)	349.3 (188.1)	333.3 (190.1)
NPI score^k^	6.3 (5.8)	5.3 (3.7)	7.1 (7.2)
CBI score^l^	10.9 (11.4)	12.1 (11.6)	9.9 (11.4)
TMS measures			
SAI, %^m^	0.79 (0.24)	0.76 (0.23)	0.81 (0.26)
Plasma markers			
Amyloid-β_1-42_ and/or amyloid-β_1-40_ level, pg/mL	0.06 (0.02)	0.06 (0.02)	0.06 (0.02)
Phosphorylated tau_217_ level, pg/mL	1.3 (0.6)	1.3 (0.7)	1.3 (0.5)
NfL level, pg/mL	25.7 (14.8)	26.8 (13.4)	24.7 (16.3)
GFAP level, pg/mL	262.8 (119.7)	260.3 (121.7)	265.1 (120.3)
**Caregivers**			
Age, y	61.1 (12.4)	59.2 (13.9)	62.9 (10.8)
Sex, No. (%)			
Female	32 (64.0)	15 (62.5)	17 (65.4)
Male	18 (36.0)	9 (37.5)	9 (34.6)
Educational level, y	13.0 (3.8)	13.1 (3.6)	12.9 (4.1)
Spouse, No. (%)	39 (78.0)	17 (70.8)	22 (84.6)

Abbreviations: AD, Alzheimer disease; ADAS-Cog-13, Alzheimer Disease Assessment Scale–cognitive subscale, 13-item version; ADCS-ADL, Alzheimer Disease Cooperative Study−Activities of Daily Living; CBI, Caregiver burden inventory; CDR-SB, Clinical Dementia Rating sum of boxes; FNAT, Face-Name Association Test; GFAP, glial fibrillary acidic protein; MMSE, Mini-Mental State Examination; NfL, neurofilament light; NPI, Neuropsychiatric Inventory; RAVL, Rey Auditory Verbal Learning Test; SAI, short-latency afferent inhibition; TMS, transcranial magnetic stimulation; TMT, Trail Making Test.

^a^ Group 1 received sham stimulation during the double-blind phase of the trial and transcranial alternating current stimulation (tACS) during the open-label phase; group 2, tACS during both treatment phases. Unless otherwise indicated, data are expressed as mean (SD).

^b^ Scores range from 0 to 30, with higher scores indicating better cognition.

^c^ Scores range from 0 to 18, with higher scores indicating greater impairment.

^d^ Scores range from 0 to 70, with higher scores indicating poorer performance.

^e^ Scores range from 0 to 78, with higher scores indicating better functioning.

^f^ Scores range from 0 to 20, with higher scores indicating better performance.

^g^ Scores range from 0 to 75, with higher scores indicating better performance.

^h^ Scores range from 0 to 30, with higher scores indicating better lexical access.

^i^ Scores range from 0 to 300, with higher scores indicating worse performance.

^j^ Scores range from 0 to 500, with higher scores indicating worse performance.

^k^ Scores range from 1 to 144, with higher scores indicating more severe neuropsychiatric symptoms.

^l^ Scores range from 0 to 96, with higher scores indicating greater caregiver burden.

^m^ Expressed as percentage of the mean value of conditioned motor-evoked potential amplitude at 0 and +4 milliseconds.

There was no association between the type of stimulation (tACS vs sham) and participants’ perception, (Cohen κ = −0.08; *P* = .57). Similarly, no association was observed for caregivers’ perception (Cohen κ = 0.17; *P* = .23), confirming effective blinding for both participants and caregivers.

Gamma tACS administered in a home setting was safe and well tolerated, with no major adverse events reported. Adherence to the intervention was high (<1.3% missed sessions) (eMethods in Supplement 2); the only adverse effect noted was light flickering, which did not impact treatment adherence. The incidence of light flickering did not differ significantly between groups during the randomized phase (group 1: 0 patients; group 2: 3 patients; *P* = .24) or the open-label phase (group 1: 1 patient; group 2: 3 patients; *P* = .61).

The perceived burden on caregivers during the 4 months of stimulation was assessed using the aforementioned 11-point Likert scale (in which 0 indicated no burden and 10 indicated high burden). The reported burden was low, with caregivers rating it as manageable (mean [SD] score, 2.6 [2.4]).

### Effects on Cognitive Functions

**Figure 2.**
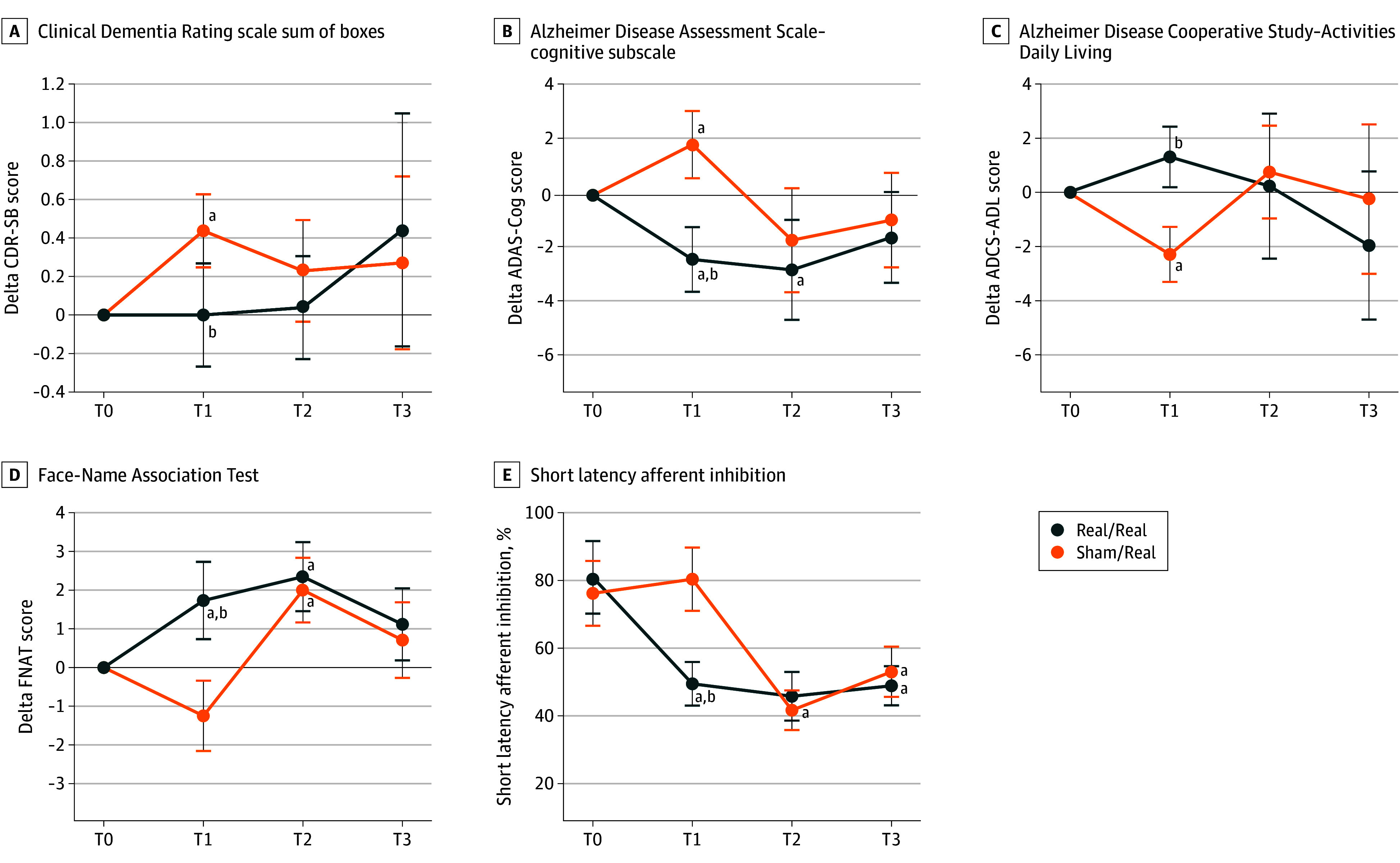
Neuropsychological Scores and Neurophysiological Measures in Groups 1 and 2 at Different Time Points Group 1 received sham stimulation during the double-blind phase of the trial (T1) and transcranial alternating current stimulation (tACS) during the open-label phase (T2); group 2 received tACS during T1 and T2. T0 indicates baseline; T3, follow-up. A-D, Scores for the Clinical Dementia Rating scale sum of boxes (CDR-SB) (A) range from 0 to 18, with higher scores indicating greater impairment; Alzheimer Disease Assessment Scale–cognitive subscale (ADAS-Cog), 13-item version (B), from 0 to 70, with higher scores indicating poorer performance; Alzheimer Disease Cooperative Study–Activities of Daily Living (ADCS-ADL) (C), from 0 to 78, with higher scores indicating better functioning; and Face-Name Association Test (FNAT) (D), from 0 to 20, with higher scores indicating better performance. E, Short latency afferent inhibition is measured as percentage of the motor-evoked potentials amplitude compared with the unconditioned motor-evoked potentials. Numeric data are given in Table 2. Error bars indicate 95% CIs. ^a^Significant difference compared with T0 after false discovery rate correction for multiple comparisons. ^b^Significant difference between groups.

Mean values of clinical variables over time for groups 1 and 2 are presented in eTable 1 in Supplement 2. A time × treatment interaction was observed for 4 indexes. In the randomized, double-blind, sham-controlled phase (T1 vs T0), marginal mean differences between groups (sham vs tACS) were significant for the CDR-SB (0.35; 95% CI, 0.10-0.61; *P* = .007), ADAS-Cog-13 (0.93; 95% CI, 0.50-1.36; *P* = .001), ADCS-ADL (−0.55; 95% CI, −0.89 to −0.21; *P* = .02), and FNAT (−1.14; 95% CI, −1.66 to −0.61; *P* < .001). During the open-label phase (T2 vs T1), a significant marginal mean difference was observed for ADAS-Cog-13 (−0.59; 95% CI, −1.02 to −0.16; *P* = .007), ADCS-ADL (0.41; 95% CI, 0.04-0.77; *P* = .02), and FNAT (1.04; 95% CI, 0.50-1.57; *P* = .003). No significant marginal mean differences were observed in the follow-up phase (T3 vs T2) (Table 2 and Figure 2).

**Table 2.** Marginal Means of Outcomes by tACS Treatment Phase

Measure	Mean (SE) score^a^		Between-groups mean difference (95% CI)	*P* value
	Group 1	Group 2		
**Randomized, double-blind phase vs baseline**				
CDR-SB	0.29 (0.09)	−0.06 (0.09)	0.35 (0.10 to 0.61)	.007
ADAS-Cog-13	0.41 (0.15)	−0.51 (0.15)	0.93 (0. 50 to 1.36)	<.001
ADCS-ADL	−0.28 (0.12)	0.27 (0.12)	−0.55 (−0.89 to −0.21)	.02
FNAT	−0.46 (0.19)	0.68 (0.18)	−1.14 (−1.66 to −0.61)	<.001
SAI	0.07 (0.19)	−1.17 (0.18)	1.25 (0.72 to 1.78)	<.001
**Open-label phase vs double-blind phase**				
CDR-SB	−0.13 (0.08)	−0.02 (0.08)	−0.16 (−0.37 to 0.06)	.71
ADAS-Cog13	−0.70 (0.15)	−0.11 (0.15)	−0.59 (−1.02 to −0.16)	.007
ADCS-ADL	−0.36 (0.13)	−0.04 (0.13)	0.41 (0.04 to 0.77)	.02
FNAT	−1.31 (0.19)	−0.27 (0.18)	1.04 (0.50 to 1.57)	.003
SAI	−1.47 (0.19)	−0.13 (0.18)	−1.34 (−1.86 to −0.83)	<.001
**Follow-up vs open-label phase**				
CDR-SB	−0.02 (0.11)	−0.11 (0.11)	−0.09 (−0.41 to 0.23)	.99
ADAS Cog13	−0.19 (0.14)	−0.25 (0.13)	−0.06 (−0.45 to 0.33)	>.99
ADCS-ADL	−0.16 (0.16)	−0.18 (0.15)	0.02 (−0.42 to 0.45)	>.99
FNAT	−0.60 (0.20)	−0.55 (0.19)	−0.05 (−0.60 to 0.50)	>.99
SAI	−0.44 (0.17)	−0.17 (0.17)	0.26 (−0.22 to 0.75)	.89

Abbreviations: ADAS-Cog-13, Alzheimer Disease Assessment Scale–cognitive subscale, 13-item version; ADCS-ADL, Alzheimer Disease Cooperative Study−Activities of Daily Living; CDR-SB, Clinical Dementia Rating sum of boxes; FNAT, Face-Name Association Test; SAI, short-latency afferent inhibition.

^a^ Group 1 received sham stimulation during the double-blind phase of the trial and transcranial alternating current stimulation (tACS) during the open-label phase; group 2, tACS during both treatment phases.

In the overall sample (groups 1 and 2 combined), significant improvements were observed in ADAS-Cog13 (0.20 to −0.05; Δ, −0.24; *P* = .04) and FNAT (−0.34 to −0.01; Δ, 0.30; *P* = .02) from T0 to T3, indicating beneficial effects of gamma tACS regardless of treatment duration (eFigure 2 in Supplement 2). Further analyses revealed that reductions in ADAS-Cog-13 scores were directly correlated with reductions in CDR-SB scores (*r* = 0.572; *P* < .001) and indirectly correlated with improvements in FNAT scores (*r* = −0.453; *P* < .001). These findings suggest that greater improvements in global cognitive function were associated with greater reductions in disease severity and better associative memory performance. No significant effects of gamma tACS were observed on episodic memory (eg, Rey Auditory Verbal Learning Test), semantic memory (eg, semantic fluency), executive functions and attention (eg, Trail Making Text), behavioral disturbances (eg, Neuropsychiatric Inventory), or caregiver burden (eg, Caregiver Burden Inventory).

### Effect on Cholinergic Neurotransmission

Mean values of SAI over time for groups 1 and 2 are presented in eTable 2 in Supplement 2. A time × treatment interaction was observed for SAI. During the randomized, double-blind, sham-controlled phase (T1 vs T0) and during the open-label phase (T2 vs T1), a significant marginal mean difference between groups (sham vs tACS) was observed for SAI (1.25 [95% CI, 0.72-1.78; *P* < .001] and −1.34 [95% CI, −1.86 to −0.83; *P* < .001], respectively). However, no significant marginal mean differences were found during the follow-up phase (T3 vs T2) (Table 2 and Figure 2).

In the overall sample (groups 1 and 2), a significant decrease in SAI was observed from T0 to T3 (0.75 to −0.31; Δ, −1.06; *P* < .001), demonstrating indirectly a restoration of cholinergic neurotransmission that was independent of treatment duration (eFigure 2 in Supplement 2). Furthermore, the restoration of SAI was indirectly correlated with improvements in associative memory, as measured by the FNAT (*r* = −0.324; *P* < .001). These results suggest that greater increases in SAI were associated with greater improvements in associative memory performance.

### Effect on Plasma Biomarkers

Gamma tACS did not induce significant effects on levels of AD-related biomarkers, including plasma levels of phosphorylated tau_217_, amyloid-β_1-42_, and amyloid-β_1-40_. Similarly, no significant changes were observed in the levels of neurofilament light, a marker of neurodegeneration, or in those of glial fibrillary acidic protein, a marker of astrogliosis.

### Effect on Precuneus Connectivity

The analysis included 27 age- and sex-matched patients with AD (group 1: 12 patients; group 2: 15 patients) who underwent MRI scanning at T0 and T1. Seed-based connectivity analysis of the precuneus region revealed no significant differences between groups (group 1 vs group 2, 5.4 vs 4.9; *P* = .26) or within groups over time (T0 vs T1, 5.2 vs 5.3; *P* = .18).

### EEG Analysis

**Figure 3.**
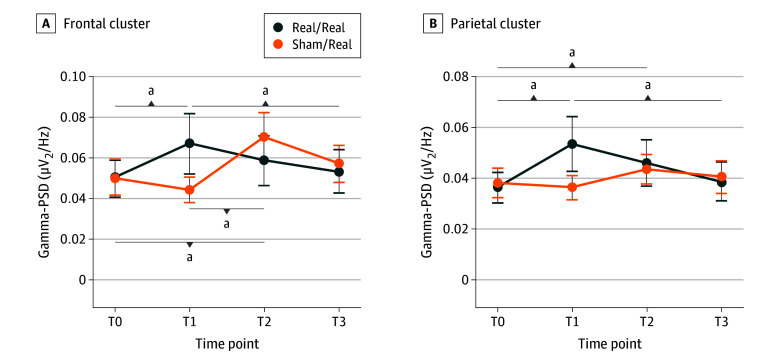
Electroencephalographic Gamma Power Spectral Density (PSD) Analysis in Groups 1 and 2 at Different Time Points in the Frontomedial and Parietal Clusters of Electrodes Group 1 received sham stimulation during the double-blind phase of the trial (T1) and transcranial alternating current stimulation (tACS) during the open-label phase (T2); group 2 received tACS during T1 and T2. T0 indicates baseline; T3, follow-up. ^a^*P* < .05.

As shown in Figure 3, analysis of power spectral density (PSD) in the parietomedial cluster revealed a significant time × treatment interaction for the only gamma frequency (*F*_3,135_ = 3.023; η^2^ = 0.063; *P* = .03), with no effects of time (*F*_3,135_ = 2.243; η^2^ = 0.047; *P* = .09) or treatment (F_1,45_ = 0.183; η^2^ = 0.004; *P* = .67). In group 2 (tACS to tACS), post hoc analyses revealed a significant increase of gamma PSD at T1 (mean [SD] difference, 0.017 [0.006 µV^2^/Hz; *t*_45_ = 3.129; *P* = .003) and at T2 (mean [SD] difference, 0.011 [0.005] µV^2^/Hz; *t*_45_ = 2.041; *P* = .047) compared with T0. We also observed a significant decrease of gamma PSD at T3 compared with T0 (mean [SD] difference, 0.015 [0.006] µV^2^/Hz; *t*_45_ = 2.561; *P* = .01).

A similar result was observed when considering the frontomedial cluster, with a significant time × treatment interaction for the only the gamma frequency (*F*_3,135_ = 3.598; η^2^ = 0.074; *P* = .02). In group 2, post hoc analyses revealed a significant increase of gamma PSD at T1 compared with T0 (mean [SD] difference, 0.016 [0.007] µV^2^/Hz; *t*_45_ = 2.289; *P* = .03) and decrease of gamma PSD at T3 compared with T1 (mean [SD] difference, 0.014 [0.006] µV^2^/Hz; *t*_45_ = 2.187; *P* = .03). Moreover, in group 1 (sham stimulation to tACS), there was a significant increase of gamma PSD at T2 compared with T0 (mean [SD] difference, 0.021 [0.011] µV^2^/Hz; t_45_ = 2.151; *P* = .04) and at T1 compared with T0 (mean [SD] difference, 0.026 [0.008] µV^2^/Hz; *t*_45_ = 3.139; *P* = .003). No significant effects were observed for the other frequency bands.

## Discussion

In this randomized, double-blind, sham-controlled clinical trial with an open extension phase, we observed that gamma tACS over the precuneus was safe and well tolerated in a home-based setting, enhancing global cognitive functions, activities of daily living, and associative memory performances. These improvements were supported by neurobiological effects on cholinergic transmission and EEG frequency modulation, specifically an increase in gamma frequencies over parietal and frontal clusters. Importantly, given that memory function strongly depends on cholinergic transmission, we found a correlation between memory performance and SAI, an indirect marker of cholinergic transmission, further supporting the role of cholinergic modulation in the observed cognitive improvement.

We also demonstrated the specificity of the stimulation parameters used in modulating brain networks, with no effects on executive functions, attention, or behavioral disturbances. Additionally, we optimized the duration of the intervention, demonstrating that an 8-week treatment did not differ significantly from a 16-week treatment at follow-up assessment, suggesting that 8 weeks may represent the optimal duration.

The lack of between-group differences during the open-label and follow-up phases should not be interpreted as evidence that initial improvements were due to chance. In the randomized phase, only participants receiving real stimulation showed consistent clinical gains and neurophysiological changes, including increased gamma power on EEG and enhanced cholinergic transmission (SAI), supporting a true biological effect. When sham-treated participants began tACS in the open-label phase, they improved similarly, while those already treated reached a plateau, suggesting a ceiling effect rather than lack of efficacy.

This study extends the recent growing body of literature supporting the usefulness of tACS interventions in AD.^7,28,29^ However, one of the main strengths of the present study is its home-based nature, which reduces the burden of a multisession treatment intervention, lowers costs associated with institutional-based care, and improves patients’ compliance. Moreover, our findings suggest that by specifically targeting and entraining brain oscillations, particularly through the normalization of gamma frequencies, tACS might indirectly stimulate cholinergic pathways, which in turn could enhance cognitive functions. Thus, gamma tACS might act not only as a direct modulator of brain rhythms but also as an indirect enhancer of neurotransmitter systems critical for cognitive function, opening avenues for multimodal therapeutic strategies that capitalize on this interaction.

In the same view, previous studies have suggested that daily light and sound at gamma frequency (GENUS) effectively induces brain entrainment in patients with mild AD, leading to a better performance in associative memory tasks and daily activities, increased functional brain connectivity, and reduced hippocampal atrophy after a 3-month intervention.^30^ Indeed, not all patients with AD are eligible for monoclonal antibody therapies due to clinical, biological, or logistical constraints. Therefore, there is a pressing need to identify alternative and sustainable treatment strategies that can be offered more broadly to patients and their families. In this context, our findings suggest that home-based gamma tACS may represent a promising nonpharmacological intervention. However, while these findings are promising, the long-term duration of the cognitive improvements post stimulation, potential biological markers of restoration, and the effects of gamma tACS on brain connectivity require further investigation.

### Limitations

This study has some limitations. First, a larger sample of participants may further strengthen the results. Second, although the home-based setting enhances feasibility and compliance, a potential limitation is the reduced direct supervision of the stimulation sessions; future studies should implement additional strategies to ensure optimal monitoring of the intervention. Third, we did not consider a longer follow-up to evaluate long-term effects after gamma tACS. Finally, future studies should consider possible modulators of treatment response, such as genetic or environmental factors, to better refine and individualize therapeutic benefits at the single-participant level.

## Conclusions

The findings of this randomized clinical trial suggest that gamma tACS over the precuneus may be safely and effectively applied in a home-based setting and is a promising approach for enhancing cognitive function and modulating neurophysiological deficits in AD. These findings contribute to the growing evidence supporting noninvasive brain stimulation techniques in neurodegenerative diseases and pave the way for further studies that could ultimately lead to the development of more effective interventions for managing AD.

## Notes

Supplement 1.
Trial Protocol

Supplement 2. **eMethods.** Inclusion and Exclusion Criteria, Cognitive Assessment, and Statistical Analysis **eTable 1.** Mean Values of Clinical Variables in Group 1 and in Group 2 **eTable 2.** Mean Values of Neurophysiological and Plasma Markers in Group 1 and in Group 2 **eFigure 1.** CONSORT Diagram **eFigure 2.** Evaluation of Variables Trend Independently in the Overall Samples (Group 1 and Group 2)
eReferences

Supplement 3.
Data Sharing Statement
