# Supplementary material for: Home-Based Gamma Transcranial Alternating Current Stimulation in Patients With Alzheimer Disease: A Randomized Clinical Trial
Source: JAMA Netw Open. 2025 Dec 8;8(12):e2546556. doi: 10.1001/jamanetworkopen.2025.46556 (PMC12687098; doi:10.1001/jamanetworkopen.2025.46556)
Supplement: Supplement 1. — Trial Protocol [file jamanetwopen-e2546556-s001.pdf]

EXPERIMENTAL CLINICAL PROTOCOL (TRANSLATION FROM ITALIAN)

TITLE OF THE STUDY: "Markers of Clinical and Biological Response to Home-based Transcranial Alternating Current Stimulation (tACS) in Patients with Alzheimer's Disease"

CODE: TACS03

VERSION AND DATE: Version 1.4

PROMOTER: ASST Spedali Civili di Brescia

PRINICIPAL INVESTIGATOR:
Dott. Stefano Gazzina
U.O. Neurologia, ASST Spedali Civili di Brescia

Other investigators involved:
Prof.ssa Barbara Borroni
Laboratorio Marcatori Molecolari, IRCCS Centro San Giovanni di Dio Fatebenefratelli di Brescia
Dipartimento di Scienze Cliniche e Sperimentali, Università degli Studi di Brescia

Prof. Alessandro Padovani
Dipartimento di Scienze Cliniche e Sperimentali, Università degli Studi di Brescia

Dott. Alberto Benussi
Dipartimento di Scienze Cliniche e Sperimentali, Università degli Studi di Brescia

Dott.ssa Valentina Cantoni
Dipartimento di Scienze Cliniche e Sperimentali, Università degli Studi di Brescia

Prof. Giacomo Koch, Università degli Studi di Ferrara
Prof. Alessandro Martorana, Policlinico Tor Vergata, Roma
Dr.ssa Chiara Cupidi, Fondazione Istituto G. Giglio, Cefalù (PA)

Dott.ssa Roberta Ghidoni, Laboratorio Marcatori Molecolari, IRCCS Centro San Giovanni di Dio Fatebenefratelli di Brescia

Dott.ssa Sonia Bellini, Laboratorio Marcatori Molecolari, IRCCS Centro San Giovanni di Dio Fatebenefratelli di Brescia

Dott. Antonio Longobardi, Laboratorio Marcatori Molecolari, IRCCS Centro San Giovanni di Dio Fatebenefratelli di Brescia

INTRODUCTION AND RATIONALE:

Brain oscillations are ubiquitous in the human brain and have been implicated in defined cognitive and behavioral states in precisely tuned neural networks. In neurodegenerative diseases, neurodegeneration is accompanied by changes in oscillatory activity, leading to the emerging concept of neurological and psychiatric disorders as "oscillopathies". Alzheimer's disease and Frontotemporal dementia, which represent the vast majority of age-related dementias, are characterized by significant alterations in brain oscillations. The restoration of oscillations through neuronal "entrainment" in animal models has shown a remarkable reduction in the neuropathological burden of toxic proteins, with a consequent significant increase in cognitive performance.

Transcranial alternating current stimulation (tACS) is a non-invasive neurophysiological technique for modulating the excitability of the central nervous system, which is gaining increasing therapeutic applications. Recent studies have demonstrated the effectiveness of this method in modulating natural brain oscillation frequencies underlying multiple cognitive processes such as verbal memory, perception, and working memory (Herrmann et al., 2013; Hoy et al., 2015).

A recent study conducted at our Center demonstrated the safety and clinical efficacy of tACS in Alzheimer's Disease, with significant improvement in memory and cholinergic circuits, which are primarily involved in the disease (see NP4479).

Based on these premises, we propose the same treatment with multiple home-based sessions in patients with mild Alzheimer's Disease to achieve a greater and more lasting effect over time. A brain stimulation system specifically designed for safe home use will be employed. The device is equipped with all safety controls to provide appropriate and safe stimulation. If inconsistencies (e.g., detached or mispositioned electrodes) or high impedances (e.g., improperly prepared electrodes) are detected, stimulation will automatically be interrupted. This procedure poses no greater risk to the patient at home than in a hospital setting and allows them to

undergo treatment at home without the need for daily hospital visits over the four-month treatment period.
To evaluate the effects on brain transmission and the underlying mechanisms of this modulation, specific intracortical circuits will be investigated using transcranial magnetic stimulation (TMS), which depends on various neurotransmitters. Over the years, numerous TMS protocols have been developed to obtain neurophysiological parameter information for investigating both the pathogenesis and progression of certain neurodegenerative diseases. Cholinergic brain activity will be assessed using the short-latency afferent inhibition (SAI) protocol (Tokimura et al., 2000). Additionally, electroencephalographic modifications and biological parameters will be analyzed.

OBJECTIVES: The aim of this study is to evaluate clinical and biological response markers to home-based transcranial alternating current stimulation (tACS) supervised via telemedicine by investigators in patients with Alzheimer's disease (AD).

PROCEDURES: Patients followed at the Neurological Clinic, ASST Spedali Civili di Brescia, and the Neurology Unit, G. Giglio Institute Foundation, Cefalù (PA), with an AD diagnosis based on current clinical criteria, will be enrolled and randomized into two groups:

The first group (group 1) will undergo stimulation protocol using tACS (real), while the second group (group 2) will undergo placebo treatment (sham) (double-blind phase), with a real:placebo ratio of 1:1. Both group 1 and group 2 will then follow an open-label phase with real tACS stimulation. The treatment will be administered during the first week in the hospital, and thereafter, it will be provided at home with supervision from one of the experimenters via telemedicine.
During the first week, the patient and caregiver will receive training at the hospital, where staff will explain how to position and use the device. On the first day, staff will show the patient and caregiver the procedures. On the following days, they will let the caregiver manage the device and will provide support during treatment administration in order to enable the caregiver to manage the device independently at home. During this period, the study's medical staff will assess compliance with the correct procedures and, therefore, the caregiver's ability to manage the device independently. At the end of the training procedure, the investigator will assess the

caregiver's ability to perform all stimulation procedures completely independently. If the caregiver is not completely independent, an additional week of training will be provided. If the caregiver is not completely independent in performing all necessary procedures independently at the end of the second week of training, the patient will be excluded from the study.

The device is easy to use, and switching it on and off, as well as the parameters, can be monitored remotely. The caregiver and patient cannot alter the set parameters, and due to the device's characteristics, they can only use the application at pre-set times and for a pre-set number of sessions, which are always set and monitored by the operator. During treatment (both sham and real), the device generates a code (reliability code) that allows the operator to know whether the procedure performed at home has been carried out correctly. If the device detects any anomalies (e.g., electrodes not positioned correctly, impedance too high), it locks itself and does not start treatment. The codes are automatically recorded by the device and can be read during the session and communicated by the patient to the operator by telephone. During the first week of home treatment, the caregiver will be asked to communicate the codes generated by the device to the center in order to verify that the procedures have been performed correctly. If the device is found to be incorrectly positioned > 50%, the caregiver will undergo further training at the center. Caregiver training at the center will be repeated a maximum of two times.

During the first week of home treatment, the patient will also be contacted ten minutes before each session by the center operator for support in managing the device. Patients and caregivers will be able to contact the investigator by telephone if necessary.

The patient will be given a copy of the device user manual (excerpt from the Italian version).

TREATMENT GROUP - GROUP 1:

The treatment group will undergo tACS sessions (real at 3 mA) applied at the cortical level for 60 minutes/day for 8 weeks, 5 days a week (Monday to Friday). The effects of tACS will be assessed both before and after stimulation, specifically evaluating cognitive performance using dedicated tests (i.e., CDR-SB, ADAS-Cog13, FNAT, ADCS-ADL), neurophysiological parameters (SAI using TMS and brain rhythms using electroencephalography [EEG]), and biological parameters (NfL, pTau, Abeta40/42, and GFAP). In a subgroup, effects on brain magnetic resonance imaging (resting state) will also be evaluated. A 6cc blood sample will also be taken to

determine the ApoE and BDNF genotype, subject to the signing of an informed consent form, in order to evaluate any markers of clinical response.

PLACEBO GROUP - GROUP 2:

In the placebo group, the setup will be the same as for real stimulation; however, the electrical current will be automatically interrupted 60 seconds after the start of each stimulation, so that the placebo stimulation is indistinguishable from the real one. The same tests and assessments used during real stimulation will be applied.

After two months of treatment, all patients will undergo an open-label phase with real tACS for an additional 2 months, following the same protocol as group 1, and a subsequent follow-up 2 months after the end of treatment. In addition, two further follow-up visits will be carried out at three months and 12 months, where only clinical evaluation will be performed.

Analysis of Biological and Genetic Markers: the following biological and genetic biomarkers of response to tACS will be analyzed:

-ApoE and BDNF Genotyping: Genomic DNA will be extracted from whole peripheral blood using the Maxwell® 16 Blood DNA Purification Kit with the Maxwell® 16 Instrument (both Promega). The regions containing both APOE polymorphisms rs429358 and rs7412, as well as BDNF rs6265, will be amplified through polymerase chain reaction (PCR) using GoTaq® Hot Start Polymerase (Promega) or Optimase® Polymerase (ADS Biotech). PCR products will be purified with 0.5 mL Amicon® Ultra centrifugal filters (Merck Millipore). Sequencing will be performed using the AB Prism Big Dye Terminator Sequencing 3.1 kit (Life Technologies), following the manufacturer's instructions. The sequences will then be purified using ID MicroSEQ™ sequencing cleanup cartridges (Life Technologies) and loaded onto a 3500 Genetic Analyzer (Life Technologies). All sequences will be analyzed using Chromas software (Technelysium Pty Ltd).

- Plasma Marker Quantification (NfL, pTau, Abeta40/42, and GFAP): plasma NfL will be measured using the commercial NF-light® assay (Quanterix, Lexington, MA) according to the manufacturer's instructions. p-Tau, Abeta40/42, and GFAP will be measured using the Simoa platform.

-Analysis of extracellular vesicles (EVs) from blood with analysis of neurodegeneration/inflammation markers (miRNomic profile). EVs will be isolated from plasma/serum using a commercial kit (Total Exosomes Isolation Kit,

Invitrogen) and analyzed using nanoparticle tracking analysis (NTA) on the NanoSight NS300 instrument (Malvern) to determine their concentration and size. miRNA expression analysis will be performed on EVs isolated from plasma/serum: small RNAs contained in circulating EVs will be extracted using commercial kits (e.g., exoRNeasy kit, Qiagen), libraries will be produced using commercial kits (e.g., QIAseq miRNA Library Kit Qiagen), and sequencing will be performed on the Illumina NextSeq2000 instrument.

INCLUSION CRITERIA for patients:

- Patients with mild Alzheimer's dementia (independent in basic and instrumental activities of daily living) according to current clinical criteria
- Signed Informed Consent Form
- Age ≥ 18 years
- Presence of a caregiver who can assist the patient and has successfully completed the necessary training for device use

EXCLUSION CRITERIA for patients:

- Individuals unable to provide informed consent
- Presence of fixed electrical stimulators (e.g., pacemakers, nerve stimulators, hearing implants) that may malfunction or be damaged by electrical or magnetic fields
- Presence of certain metallic foreign bodies (e.g., intrauterine devices, shrapnel, some prostheses, screws, and nails) that could move within a magnetic field
- History of epilepsy
- Pregnant women, as the effects of electrical or magnetic stimulation on a developing fetus are unknown

INCLUSION CRITERIA for the caregiver:

- Signed Informed Consent Form
- Age > 18 years
- Compliance in participating in training on device usage
- Mini-Mental State Examination (MMSE) > 27/30

EARLY WITHDRAWAL CRITERIA from the study:

- Difficulty in managing the device, measured by the procedure failure rate, as indicated by the reliability code generated by the device after two training sessions at the center.

STUDY DESIGN: it is a monocentric, interventional, non-pharmacological, placebo-controlled, randomized, double-blind clinical study, as neither the patients nor those administering the evaluation tests will be aware of the type of treatment provided, real tACS or placebo, followed by an open-label phase. The stimulation equipment will be pre-programmed (real stimulation or placebo) by another operator who will not administer the stimulation itself or the tests, thus ensuring the experimental double-blind. Both the operator administering the treatment and the operator performing the evaluations will be unaware of the type of stimulation administered.
At baseline (T0), at the end of the double-blind phase (T1, 8 weeks), at the end of the open-label phase (T2, 16 weeks), and after further follow-up (T3, 24 weeks), each subject will undergo clinical and neuropsychological evaluation, TMS, EEG, and blood sampling for the assessment of biological markers such as NfL, pTau, Abeta40/42, and GFAP. The biological samples will then be sent to the Molecular Markers Laboratory at the IRCCS Istituto Centro San Giovanni di Dio Fatebenefratelli in Brescia for analysis of biological markers on extracellular vesicles from plasma or serum.
A subgroup of patients will undergo brain MRI (resting state) at baseline (T0) and at the end of the double-blind phase (T1, 8 weeks).
Each subject will then undergo two further follow-up visits with clinical evaluation at 36 weeks (T4) and 72 weeks (T5) from T0.

OBJECTIVES:
- Primary objectives: evaluate the tolerability and safety of transcranial alternating current stimulation (tACS) and its effectiveness on dedicated tests (CDR-SB, ADAS-Cog13, FNAT, ADCS-ADL).
- Secondary objectives: evaluate the effectiveness of tACS on neurophysiological imaging parameters, markers of neurodegeneration/plasma amyloidosis.

- Exploratory objectives: evaluate clinical and biological markers at baseline (cognitive reserve index questionnaire, ApoE genotype, BDNF) in response to tACS.

END POINTS:
- Primary objectives: it is expected that tACS will be well tolerated (% of compliance >50%) and safe (absence of major adverse events) in the studied population. The effectiveness of the treatment will be evaluated on dedicated tests.
- Secondary objectives: the effectiveness of the treatment on imaging parameters will be evaluated. The effectiveness of the treatment on neurophysiological parameters and plasma neurodegeneration/amyloidosis will be evaluated.
- Exploratory objectives: it is expected that certain biological factors at baseline (e.g., ApoE genotype) may modulate the degree of response to tACS and thus predict the degree of clinical response.

STATISTICAL PLAN: as an experimental design, we will use a linear mixed-effects model (LME) with TIME (T0, T1, T2, T3) as a within-subject factor and TREATMENT (sham/real vs real/real) as between-subject factors, considering the subject modeled as a random intercept effect.
We performed a power analysis with G*Power to estimate the sample size, based on some previously published studies (Benussi et al., *Brain Stimul* 2021; Dhaynaut et al., *J Alzheimers Dis* 2022; Zhou et al., *J Neurol Neurosurg Psychiatry* 2021), using methodologies similar to those applied in the study in question. The effect size f(V) was calculated using the direct method. Taking into account from the aforementioned studies an effect size equal to a partial η^2 of 0.080, corresponding to an effect size f(V) of 0.295, and considering alpha = 0.05 and power (1-β) = 0.90, the expected sample size is 34 total subjects. Taking into account a possible attrition rate of 50%, appropriate for long-term studies in patients with Alzheimer's disease, who may have poor tolerability to the device, the final required sample size amounts to 50 total subjects (25 per group).
Correlation analyses (Pearson correlations corrected for multiple comparisons with False Discovery Rate) will be used to evaluate the correlations between biological parameters (plasma neurodegeneration markers) and clinical response. For dichotomous measures (ApoE and BDNF genotype), Fisher's exact test will be used.

ETHICAL CONSIDERATIONS (RISK-BENEFIT): tACS is a non-invasive, low-cost, and easy-to-implement technique that is gaining increasing popularity and an expanding range of potential therapeutic applications.

The tACS used in this study (model: REMOTE mini-CT tES; company: Soterix Medical Inc.) is a medical device with CE marking (no. G1 089785 0013 rev. 00). We hypothesize that tACS may have a positive effect on naming performance in patients with neurodegenerative diseases, such as Alzheimer's disease, and that tACS may have a positive effect on the normalization of neurophysiological parameters that are altered in individuals affected by such neurodegenerative diseases, for which no treatment currently exists.
Literature data show that the application of this method, according to the current safety guidelines (Iyer, 2005; Nitsche, 2003; Wassermann 2005; Antal 2017), has minimal side effects, including mild tingling sensations during or after stimulation, moderate fatigue, mild itching at the electrode application sites, mild burning or pain sensations, headache, nervousness, nausea, and the appearance of vesicles at the electrode application sites in rarer cases.
The stimulation performed at home will not expose the patient to a higher risk compared to stimulation in a hospital setting. The device is designed to be used at home and is equipped with all the safety systems to prevent incorrect or potentially dangerous stimulations.
Regarding the TMS technique, it is a non-invasive brain stimulation method that allows the study of neurophysiological parameters, which can provide information on the pathophysiological mechanisms involved in neurodegenerative diseases.
Literature data show that the application of the method according to current safety guidelines has minimal side effects: a certain number of subjects participating in TMS experiments (up to 20%) report headaches or back pain, most likely due to excessive muscle tension and a rigid position of the head and/or neck during TMS application. These effects are temporary and, in most cases, do not require any treatment.
Since TMS produces a magnetic field, individuals with implanted electrical stimulators (e.g., pacemakers, nerve stimulators, cochlear implants) who would not function or could be damaged by the magnetic field cannot participate in the study. Also excluded are individuals with specific metallic foreign bodies (e.g., shrapnel, certain prosthetics, screws, and nails) that could move if placed within the magnetic field. Since the effects of TMS on the developing fetus are not known, pregnant women cannot participate in the study.

PERSONAL DATA PROCESSING:

The data will be processed in compliance with Legislative Decree 196/2003 and subsequent authorizations and amendments, as well as with European Regulation No.

679/2016 concerning privacy protection, and will be used for the purposes of the study.

ADVERSE EVENTS MONITORING:

It will be the responsibility of the Principal Investigator to promptly notify the CET6 of any adverse event induced by the method.

REFERENCES:

Alberici A, Bonato C, Calabria M, et al. (2008) The contribution of TMS to frontotemporal dementia variants. Acta Neurol Scand 118:275–280. doi: 10.1111/j.1600-0404.2008.01017.x

Antal, A, I Alekseichuk, M Bikson, J Brockmöller, A R Brunoni, R Chen, L G Cohen, et al. "Low Intensity Transcranial Electric Stimulation: Safety, Ethical, Legal Regulatory and Application Guidelines." Clinical Neurophysiology 128, no. 9 (September 2017): 1774–1809.

Asmussen MJ, Jacobs MF, Lee KGH, et al. (2013) Short-latency afferent inhibition modulation during finger movement. PLoS ONE 8:e60496. doi: 10.1371/journal.pone.0060496

Benussi A, Cantoni V, Cotelli MS, Cotelli M, Brattini C, Datta A, et al. Exposure to gamma tACS in Alzheimer's disease: a randomized, double-blind, sham-controlled, crossover, pilot study. Brain Stimul. 2021;14.

Dhaynaut M, Sprugnoli G, Cappon D, Macone J, Sanchez JS, Normandin MD, et al. Impact of 40 Hz Transcranial Alternating Current Stimulation on Cerebral Tau Burden in Patients with Alzheimer's Disease: A Case Series. J Alzheimers Dis. 2022;85:1667–76.

Di Lazzaro V, Pilato F, Dileone M, et al. (2007) Segregating two inhibitory circuits in human motor cortex at the level of GABAA receptor subtypes: a TMS study. Clin Neurophysiol 118:2207–2214. doi: 10.1016/j.clinph.2007.07.005

Freitas C, Mondragón-Llorca H, Pascual-Leone A (2011) Noninvasive brain stimulation in Alzheimer's disease: systematic review and perspectives for the future. Exp Gerontol 46:611–627. doi: 10.1016/j.exger.2011.04.001

Herrmann CS, Rach S, Neuling T, Struber D. Transcranial alternating current stimulation: a review of the underlying mechanisms and modulation of cognitive process. Review article. Frontiers in human neuroscience 2013- vol, 7 – article 279.

Herrmann CS, Rach S, Neuling T, Struber D. Transcranial alternating current stimulation: a review of the underlying mechanisms and modulation of cognitive process. Review article. Frontiers in human neuroscience 2013- vol, 7 – article 279.

Hoy KE, Bailey N, Arnold S, Windsor K, John J, Daskalakis ZJ, Fitzgerald PB. The effect of γ- tACS on working memory performance in healthy controls. Brain and Cognition 101 (2015) 51-56.

Jacobs M, Premji A, Nelson AJ (2012) Plasticity-Inducing TMS Protocols to Investigate Somatosensory Control of Hand Function. Neural Plasticity 2012:1–12. doi: 10.1155/2012/350574

Koch G, Esposito Z, Codecà C, et al. (2011) Altered dopamine modulation of LTD-like plasticity in Alzheimer's disease patients. Clin Neurophysiol 122:703–707. doi: 10.1016/j.clinph.2010.10.033

Koch G, Esposito Z, Kusayanagi H, et al. (2011) CSF tau levels influence cortical plasticity in Alzheimer's disease patients. J Alzheimers Dis 26:181–186. doi: 10.3233/JAD-2011-110116

Nitsche MA, Cohen LG, Wassermann EM, Priori A, Lang N, Antal A, Paulus W, Hummel F, Boggio PS, Fregni F, Pascual-Leone A. Transcranial direct current stimulation: State of the art 2008. Brain Stimul. 2008 Jul;1(3):206-23. Epub 2008 Jul 1. Review.

Pierantozzi M, Panella M, Palmieri MG, et al. (2004) Different TMS patterns of intracortical inhibition in early onset Alzheimer dementia and frontotemporal dementia. Clinical Neurophysiology 115:2410–2418. doi: 10.1016/j.clinph.2004.04.022

Tokimura H, Di Lazzaro V, Tokimura Y, et al. (2000) Short latency inhibition of human hand motor cortex by somatosensory input from the hand. J Physiol (Lond) 523 Pt 2:503–513. doi: 10.1111/j.1469-7793.2000.t01-1-00503.x

Zhou D, Li A, Li X, Zhuang W, Liang Y, Zheng C-Y, et al. Effects of 40 Hz transcranial alternating current stimulation (tACS) on cognitive functions of patients with Alzheimer's disease: a randomised, double-blind, sham-controlled clinical trial. J Neurol Neurosurg Psychiatry. 2021;jnnp-2021-326885.
