# Supplementary material for: Home-Based Gamma Transcranial Alternating Current Stimulation in Patients With Alzheimer Disease: A Randomized Clinical Trial
Source: JAMA Netw Open. 2025 Dec 8;8(12):e2546556. doi: 10.1001/jamanetworkopen.2025.46556 (PMC12687098; doi:10.1001/jamanetworkopen.2025.46556)
Supplement: Supplement 2. — eMethods. Inclusion and Exclusion Criteria, Cognitive Assessment, and Statistical Analysis eTable 1. Mean Values of Clinical Variables in Group 1 and in Group 2 eTable 2. Mean Values of Neurophysiological and Plasma Markers in Group 1 and in Group 2 eFigure 1. CONSORT Diagram eFigure 2. Evaluation of Variables Trend Independently in the Overall Samples (Group 1 and Group 2) eReferences [file jamanetwopen-e2546556-s002.pdf]

Supplementary Online Content

Cantoni V, Casula EP, Tarantino B, et al. Home-based gamma transcranial alternating current stimulation in patients with Alzheimer disease: a randomized clinical trial. *JAMA Netw Open.* 2025;8(12):e2546556. doi:10.1001/jamanetworkopen.2025.46556

eMethods. Inclusion and Exclusion Criteria, Cognitive Assessment, and Statistical Analysis
eTable 1. Mean Values of Clinical Variables in Group 1 and in Group 2
eTable 2. Mean Values of Neurophysiological and Plasma Markers in Group 1 and in Group 2
eFigure 1. CONSORT Diagram
eFigure 2. Evaluation of Variables Trend Independently in the Overall Samples (Group 1 and Group 2)
eReferences

This supplementary material has been provided by the authors to give readers additional information about their work.

© 2025 Cantoni V et al. *JAMA Network Open*.

eMethods. Inclusion and Exclusion Criteria, Cognitive Assessment, and Statistical Analysis

Inclusion and exclusion criteria.

Inclusion criteria were the following: *a)* biologically confirmed diagnosis of AD (according to pattern of cerebrospinal amyloid-β_{1-42}, tau and p-tau_{181} dosages or positive PET amyloid imaging; *b)* prodromal or mild AD (Clinical Dementia Rating Scale = 0.5 or Clinical Dementia Rating Scale=1, respectively); *c)* participants on a stable pharmacological regimen for at least six weeks prior to the intervention continued their treatment; no new medications were allowed during the study.

Exclusion criteria were the following: *a)* other causes of cognitive deficits other than AD; *b)* history of head injury, transient ischemic attack, stroke, epilepsy, or medical disorders causing cognitive decline; *c*) presence of cardiac devices or prior brain surgery; *d)* absence of a compliant caregiver.

Cognitive assessment.

At baseline and at each time point, cognitive and behavioral assessment was carried out.

To assess global cognitive functions, we used the Clinical Dementia Rating – Sum of Boxes (CDR-SOB) and the Alzheimer's Disease Assessment Scale – Cognitive Subscale (ADAS-Cog13) rating scales. The CDR covers six domains and is based on a semi-structured interview with the caregiver. The CDR yields both a global score (0–3) and a CDR-SOB score (0–18) representing the sum score of the six domain ratings. Higher scores indicate greater severity/impairment.[1] The ADAS-Cog13 is a performance-based assessment of cognitive functions which includes 13 tasks; the ADAS-Cog 13 total score ranges from 0–85.[2]

To assess associative memory, The Face-Name Association memory Task (FNAT) was used, which was composed of encoding and retrieval phases, as previously described.[3] The stimuli were presented using Presentation software (Version 14.9, www.neurobs.com). During the encoding phase, the patient was shown a gray-scale picture of a face on a monitor together with a proper name, and the patient was required to encode the face-name association. A set of 20 unfamiliar faces was associated to a set of 20 unfamiliar proper names (10 male, 10 female). During the retrieval phase, the patient was shown a face together with four proper names (the correct name, two previously presented names and one new name), and the patient was asked to associate the correct name with each face. Responses were collected via a response-box, and the stimuli remained on the screen until the response was made. Different sets of stimuli were randomized to avoid learning effects.

© 2025 Cantoni V et al. *JAMA Network Open*.

To assess episodic memory, the Rey auditory verbal learning (RAVL) test was carried out, and immediate and delayed recall scores were recorded.[4] Different lists were randomized and used to avoid learning effects.

To assess semantic memory, we considered semantic fluency, which is scored by counting the number of correct unique semantic categories produced.[5]

To assess executive functions and attention, the trail making test part A (TMT-A) and B (TMT-B) were used, using 4 different versions to avoid learning effects.[6]

To assess activities of daily living, we used the Alzheimer's Disease Cooperative Study-Activities of Daily Living (ADCS-ADL) scale, which relies on 23 basic and instrumental ADLs with information provided by the caregiver. Each activity is scored ranging from 0 (patient does not perform the activity) to the highest score (patient is independent in the activity).[7]

To evaluate behavioral and neuropsychiatry symptoms, we adopted the Neuropsychiatry Inventory (NPI), which rates the presence and the severity of behavioral changes based on responses from the caregiver.[8] To evaluate caregiver burden, we administered the Caregiver Burden Inventory (CBI).[9]

Transcranial magnetic stimulation assessment.

A TMS figure-of-eight coil (each loop diameter 70 mm) connected to a monophasic Magstim Bistim^2 system (Magstim Company, Oxford, UK) was employed.[10] Motor evoked potentials (MEPs) were recorded from the right first dorsal interosseous muscle through surface Ag/AgCl electrodes placed in a belly-tendon montage and acquired using a Biopac MP-150 electromyograph (BIOPAC Systems Inc., Santa Barbara, USA). The TMS coil was held tangentially over the scalp region corresponding to the primary hand motor area contralateral to the target muscle, with the coil handle pointed 45° posteriorly and laterally to the sagittal plane. The "hot spot" was defined as the scalp location from which magnetic stimulation resulted in motor evoked potentials (MEPs) of greatest amplitude with the minimum stimulation intensity, as previously reported.[11]

Short latency afferent inhibition (SAI), an indirect marker of cholinergic transmission, was studied using a paired-pulse technique, employing a conditioning-test design. The test stimulus (TS) was adjusted to evoke a MEP of approximately 1 mV amplitude.

SAI was evaluated employing a conditioning stimulus (CS) of single pulses (200 µs) of electrical stimulation delivered to right median nerve at the wrist, using a bipolar electrode with the cathode positioned proximally, at an intensity sufficient to evoke a visible twitch of the thenar muscles.[12] Different interstimulus intervals (ISIs) were implemented (0, +4), which were fixed relative to the N20 component latency of the somatosensory evoked potential of the median nerve.

© 2025 Cantoni V et al. *JAMA Network Open*.

For each ISI and for each protocol, ten different paired CS-TS stimuli and fourteen control TS stimuli were delivered in all participants in a pseudo-randomized sequence, with an inter-trial interval of 5 secs (±10%).

The conditioned MEP amplitude, evoked after delivering a paired CS-TS stimulus, was expressed as percentage of the average control MEP amplitude. Average values for SAI (0, +4 ms ISI) were used for analysis.

Audio-visual feedback was provided to ensure muscle relaxation during the entire experiment and trials were discarded if EMG activity exceeded 100 µV prior to TMS stimulus delivery. Less than 5% of trials were discarded for each protocol. All of the participants were capable of following instructions and reaching complete muscle relaxation.

Brain Magnetic Resonance Imaging (MRI) acquisition, preprocessing, quality assurance and analysis. A Skyra 3T clinical MR scanner (Siemens Healthcare, Erlangen, Germany) with a 64-channel head-neck RF receive coil and parallel transmit RF (operating system VE11C) was used to collect the MR data. The structural image consisted of a T1-weighted MPRAGE (TR=2s, TE=2.85ms, TI=850 ms, 1.1 mm-isotropic voxels). 200 whole-brain volumes (TR=2.5s, TE=30 ms, 3x3x3.5 mm, complete brain coverage) were obtained for the resting state run using functional BOLD 2D Echo-Planar Imaging.

A standard preprocessing pipeline was performed using the FSL software.[13] The preprocessing includes: (1) slice timing correction; (2) T1-weighted image tissue segmentation; (3) geometric distortion and head motion correction; (4) co-registration of the T1-weighted image to the time-series, (5) nuisance regressions of the mean white matter signal, the mean cerebrospinal fluid signal and the six head motion parameters; (6) band-pass temporal filtering [0.01-0.1 Hz]; (7) normalization to standard MNI template space; and (8) spatial smoothing 6 mm FWHM Gaussian kernel size. In order to check the quality of pre-processed data, the head motion during each run was assessed by computing both the framewise displacement (FD)[14] and DVARS.[15]

Following the selection of the precuneus from the anatomical Harvard-Oxford atlas[16] as the brain region to be seeded, we carried out a Region of Interest (ROI)-to-whole brain analysis. The FSL software was used to conduct the analysis, and the result was a 4D concatenation of the connectivity map for each participant. The latter were used as input for a two-way ANOVA to model variations between Group 1 and Group 2 (sham vs. real) as well as within-subjects differences (T0 - T1).

EEG recordings and analyses. In a sound-proof, temperature-controlled room, resting condition eyes-open EEG data were recorded using an actiCAP slim 64-channel active electrode system connected to an actiCHamp Plus 64 System amplifier (Brain Products, Gilching, Germany). Electrode impedance was kept below 5 kΩ. All data were online digitized (256 Hz sampling rate) and stored on a personal computer. Both artefact detection and quantitative analyses were then

© 2025 Cantoni V et al. *JAMA Network Open*.

performed offline. During the recording sessions, the participants' state of vigilance was controlled by visual inspection of EEG traces and subjects' drowsiness (i.e., slowing of EEG, slow eye movements, appearance of sleep spindles and/or K complexes, etc.). Resting EEG was recorded for 10 min at each time point.

Pre-processing and analysis was performed as follows. As a first step, data was band-pass filtered between 1 and 80 Hz (Butterworth zero phase filters). A 50 Hz notch filter was also applied to reduce noise from electrical sources. Identification and removal of artifacts (muscle activity, eye movements and blink-related activity) was made with independent component analysis (INFOMAX-ICA). Then, the signal was segmented in 2-sec epochs. Power spectral density (PSD) was estimated by means of the Fast Fourier Transform (10% Hanning-window) from 1 to 50 Hz, with a frequency resolution of 0.5 Hz. Frequency bands were established as follows: delta (1-4 Hz); theta (4-7 Hz); alpha (7-13 Hz); beta (13-30 Hz); gamma (30-50 Hz). As a final step, power density was averaged among the epochs. For our analysis we considered two clusters of 4 electrodes: one parieto-medial surrounding Pz (Pz, P1, P2, POz), which was our site of stimulation; one fronto-medial surrounding Fz (Fz, F1, F2, AFz), to monitor possible changes through the fronto-parietal connections.

Statistical analyses. For patients with missing values at follow-up (8/200 time points), data were assigned using mean values without any *ad hoc* imputation for both clinical and neurophysiological measures. Cohen's Kappa was run to determine if there was agreement between the type of sensation perceived and the type of stimulation received.

To assess the effect of tACS treatment on clinical, biological and neurophysiological measures over time, we used a mixed model (LME) (see Supplementary Materials for details).[16] The TIME variable (T0, T1, T2, T3) was modelled as a categorical factor, and TREATMENT (sham/real stimulation vs real/real stimulation) was included as a between-subject factor. The model also included a random intercept for subjects to account for within-subject correlation across repeated measurements. The interaction between TIME×TREATMENT was specified as a fixed effect to evaluate whether the effect of TREATMENT differed across the three study phases: the randomized, double-blind phase (T1 vs T0), the open-label phase (T2 vs T1), and the follow-up phase (T3 vs T2).

Two-sided *p*-values<0.05 were considered statistically significant. Fixed effect coefficients and their 95% confidence intervals (95% CIs) were reported using marginal mean differences when the interaction terms were significant, or conditional mean differences for main effects.

As exploratory analysis, we tested the associations between the improvement in clinical scores, neurophysiological parameters, and demographic or clinical characteristics. Specifically, we analysed mixed effects models considering only the TIME effect contribution on the previous response variables and we extracted the model predictions. Then, the

correlation between the TIME model predictions has been tested to assess if there was an association between the variables, using a covariance selection model.[17]

EEG data were analyzed with a two-way ANOVA with TIME and TREATMENT as fixed factors, separately for each frequency band and for the two clusters (parietal and frontal). Exploratory post-hoc analyses were performed with paired or unpaired t-tests comparing all the experimental conditions resulting from the omnibus effects.

Statistical analyses were performed with R version 4.2.2 (R Foundation for Statistical Computing, Vienna, Austria).

Adherence to treatment. Adherence to the tACS intervention was high. Each participant was scheduled to undergo 80 stimulation sessions—40 during the double-blind phase and 40 during the open-label phase. Out of a total of 4.000 scheduled sessions for the 50 enrolled participants (80 × 50 = 4.000), only 50 sessions were missed overall, corresponding to less than 1.5% of all sessions. On average, participants completed 39.6 ± 1.0 sessions in the double-blind phase and 39.6 ± 1.4 sessions in the open-label phase. Missed sessions were rare, evenly distributed across the two phases, and not associated with treatment outcomes.

© 2025 Cantoni V et al. *JAMA Network Open*.

eTable 1. Mean Values of Clinical Variables in Group 1 and in Group 2

Group	Randomized double-blind phase		Open label phase	
	**T0**	**T1**	**T2**	**T3**
**Group 1 (sham/real tACS)**				
Clinical assessment				
CDR-SB	2.8 (1.6)	3.2 (1.7)*	3.0 (1.7)	3.0 (1.8)
ADAS-Cog13	17.8 (4.7)	19.6 (4.4)*†	16.0 (5.2)	16.2 (5.2)
ADCS-ADL	65.2 (9.0)	62.9 (9.8)*†	65.9 (9.0)	63.8 (10.6)
FNAT	7.4 (2.6)	6.2 (2.0)*	9.4 (2.5)†	8.1 (2.0)
RAVL, immediate recall	20.4 (8.5)	17.3 (6.1)*†	23.0 (8.7)	23.2 (8.8)
RAVL, delayed recall	1.5 (2.1)	0.8 (1.3)	2.0 (2.1)	1.9 (2.6)
Semantic fluency	14.8 (5.1)	13.2 (5.4)*	14.8 (6.0)	14.4 (5.2)
TMT, A	81.4 (70.3)	87.4 (74.9)	81.9 (74.4)	70.7 (58.0)
TMT, B	349.3 (188.2)	393.5 (176.5)*	371.5 (178.8)	346.2 (191.0)
NPI	5.3 (3.7)	6.2 (5.0)*	5.3 (5.3)	6.1 (6.2)
CBI	12.1 (11.6)	13.5 (12.6)	15.3 (14.3)	15.3 (15.5)
**Group 2 (real/real tACS)**				
Clinical assessment				
CDR-SB	2.3 (1.2)	2.3 (1.5)*	2.4 (1.6)	2.8 (2.3)
ADAS-Cog13	17.9 (5.4)	15.4 (5.4)*†	15.0 (5.4)†	16.1 (4.9)
ADCS-ADL	67.2 (6.7)	68.5 (7.7)*	67.4 (9.9)	65.0 (10.9)
FNAT	7.1 (2.2)	8.8 (2.4)*†	9.5 (2.2)†	8.2 (2.0)
RAVL, immediate recall	22.7 (10.1)	23.3 (9.7)*	25.2 (10.2)	24.2 (9.7)
RAVL, delayed recall	2.0 (2.0)	2.1 (2.5)	2.3 (2.6)	1.8 (2.1)
Semantic fluency	14.7 (4.9)	15.1 (5.4)*	15.3 (6.2)	14.2 (6.2)
TMT, A	68.0 (57.4)	76.6 (70.9)	68.5 (56.9)	81.2 (73.4)
TMT, B	333.3 (190.1)	295.6 (186.1)*	281.4 (180.2)	297.2 (191.4)
NPI	7.1 (7.2)	6.1 (6.7)*	5.4 (5.5)	5.7 (6.9)
CBI	9.9 (11.3)	13.2 (13.9)	13.6 (15.2)	15.8 (19.1)

tACS: transcranial alternating current stimulation; CDR-SB: Clinical Dementia Rating, sum of boxes score; ADAS-Cog13: Alzheimer's Disease Assessment Scale – Cognitive Subscale, ADAS-Cog; ADCS-ADL: Alzheimer’s Disease Cooperative Study-Activities of Daily Living; FNAT: face-name association task; RAVL: Rey auditory verbal learning; TMT: trail making test; NPI: Neuropsychiatric Inventory; CBI: Caregiver burden inventory.

*significant difference between groups, considering delta scores from baseline; †significant difference compared to baseline (T0), after FDR correction for multiple comparisons.

© 2025 Cantoni V et al. *JAMA Network Open*.

eTable 2. Mean Values of Neurophysiological and Plasma Markers in Group 1 and in Group 2

Group	Randomized double-blind phase		Open label phase	
	**T0**	**T1**	**T2**	**T3**
**Group 1 (sham/real tACS)**				
TMS measures				
SAI	0.76 (0.23)	0.80 (0.22)	0.42 (0.14)†	0.53 (0.18†
Plasma markers				
amyloid-$\beta_{1\text{-}42}$/amyloid-$\beta_{1\text{-}40}$	0.1 (0.0)	0.1 (0.0)	0.1 (0.0)	0.1 (0.0)
p-tau$_{217}$, pg/mL	1.4 (0.7)	1.5 (1.0)	1.3 (0.8)	1.5 (0.6)
NfL, pg/mL	26.8 (13.4)	24.8 (11.7)	24.3 (9.8)	27.5 (16.6)
GFAP, pg/mL	260.3 (121.7)	264.3 (89.6)	260.1 (114.0)	297.1 (203.5)
**Group 2 (real/real tACS)**				
TMS measures				
SAI	0.81 (0.27)	0.49 (0.16)*†	0.46 (0.18)†	0.49 (0.14)†
Plasma markers				
amyloid-$\beta_{1\text{-}42}$/amyloid-$\beta_{1\text{-}40}$	0.1 (0.0)	0.1 (0.0)	0.1 (0.0)	0.1 (0.0)
p-tau$_{217}$, pg/mL	1.3 (0.5)	1.2 (0.4)	1.3 (0.5)	1.3 (0.6)
NfL, pg/mL	24.7 (16.3)	21.1 (10.7)	22.6 (11.9)	26.3 (16.0)
GFAP, pg/mL	265.1 (120.3)	245.8 (105.5)	252.5 (112.1)	257.5 (119.6)

tACS: transcranial alternating current stimulation; TMS: transcranial magnetic stimulation; SAI: short-latency afferent inhibition; p-tau: phospho-Tau; NfL: neurofilament light; GFAP: glial fibrillary acidic protein.

*significant difference between groups, considering delta scores from baseline; †significant difference compared to baseline (T0), after FDR correction for multiple comparisons.

© 2025 Cantoni V et al. *JAMA Network Open*.

eFigure 1. CONSORT Diagram

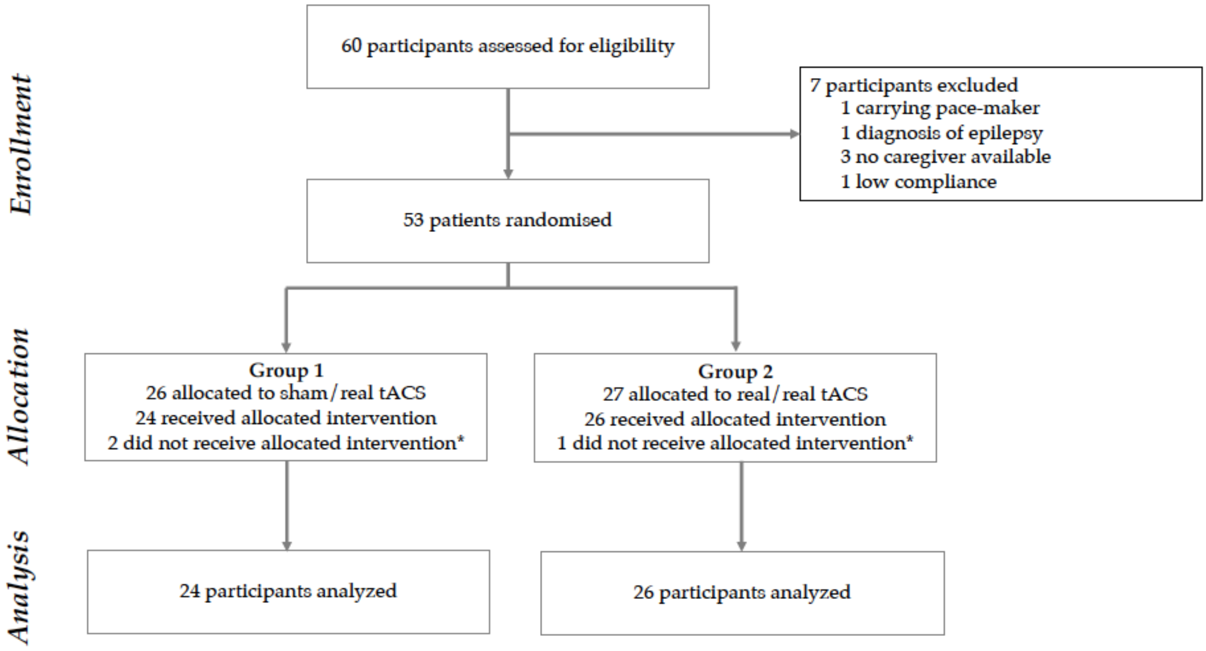

Legend: tACS= transcranial alternate current stimulation;
*1 drop-out for stroke (group 1, T0-T1), 1 for new diagnosis of cancer (group 1, T0-T1), 1 for low caregiver compliance (group 2, T0-T1)

© 2025 Cantoni V et al. *JAMA Network Open*.

eFigure 2. Evaluation of Variables Trend Independently in the Overall Samples (Group 1 and Group 2)

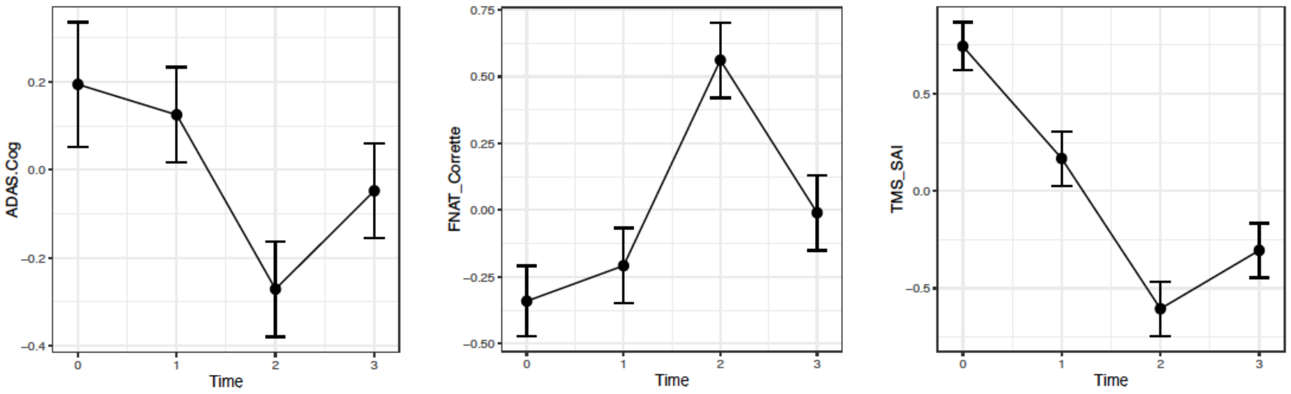

*TIME variable was evaluated in combination to its quadratic term to evaluate a linear trend and U-shaped time relationship in the overall sample.

© 2025 Cantoni V et al. *JAMA Network Open*.

eReferences

1. Williams MM, Storandt M, Roe CM, Morris JC. Progression of Alzheimer's disease as measured by Clinical Dementia Rating Sum of Boxes scores. Alzheimers Dement. 2013;9(1 Suppl):S39-44. doi: 10.1016/j.jalz.2012.01.005.
2. Verma N, Beretvas SN, Pascual B, et al. New scoring methodology improves the sensitivity of the Alzheimer's Disease Assessment Scale-Cognitive subscale (ADAS-Cog) in clinical trials. Alzheimers Res Ther. 2015;7(1):64. doi: 10.1186/s13195-015-0151-0.
3. Benussi A, Cantoni V, Grassi M, et al. Increasing Brain Gamma Activity Improves Episodic Memory and Restores Cholinergic Dysfunction in Alzheimer's Disease. Ann Neurol. 2022;92(2):322–34. doi: 10.1002/ana.26411.
4. Rey A. L'Examen Clinique en Psychologie. Clinical Examination in Psychology. 1964.
5. Novelli G, Papagno C, Capitani E, et al. Three clinical tests to research and rate the lexical performance of normal subjects [in Italian]. Arch Psicol Neurol Psichiatr. 1986;47:477–506.
6. Corrigan JD, Hinkeldey MS. Relationships between parts A and B of the Trail Making Test. J Clin Psychol 1987;43(4):402–409.
7. Galasko D, Bennett D, Sano M, et al. An inventory to assess activities of daily living for clinical trials in Alzheimer's disease. The Alzheimer's Disease Cooperative Study. Alzheimer Dis Assoc Disord. 1997; 11(Suppl 2), S33-39.
8. Cummings JL, Mega M, Gray K, et al. The Neuropsychiatric Inventory: comprehensive assessment of psychopathology in dementia. Neurology. 1994; 44(12):2308-14. doi: 10.1212/wnl.44.12.2308.
9. Novak M, Guest C. Application of a Multidimensional Caregiver. Gerontologist. 1989; 29(6):798-803. doi: 10.1093/geront/29.6.798.
10. Bracca V, Cantoni V, Gadola Y, et al. Neurophysiological correlates of altered time awareness in Alzheimer's disease and frontotemporal dementia. Neurol Sci. 2023;44(10):3515-3522. doi: 10.1007/s10072-023-06877-8.
11. Benussi A, Premi E, Cantoni V, et al. Cortical Inhibitory Imbalance in Functional Paralysis. Front Hum Neurosci. 2020; 7:14:153. doi: 10.3389/fnhum.2020.00153.
12. Tokimura H, Di Lazzaro V, Tokimura Y, et al. Short latency inhibition of human hand motor cortex by somatosensory input from the hand. J Physiol. 2000 ;523 Pt 2(2):503–13. doi: 10.1111/j.1469-7793.2000.t01-1-00503.x.
13. Jenkinson M, Beckmann CF, Behrens TE, Woolrich MW, Smith SM. FSL. NeuroImage. 2012; 62 (2), 782–790. doi: 10.1016/j.neuroimage.2011.09.015.
14. Power JD, Barnes KA, Snyder AZ, Schlaggar BL, Petersen SE. Spurious but systematic correlations in functional connectivity MRI networks arise from subject motion. 2012;59(3):2142-54. doi: 10.1016/j.neuroimage.2011.10.018.
15. Afyouni S, Nichols TE. Insight and inference for DVARS. NeuroImage. 2018; 15:172:291-312. doi: 10.1016/j.neuroimage.2017.12.098.
16. Kennedy D, Haselgrove C, Fischl B, et al. Harvard-Oxford cortical and subcortical structural atlases. Harvard Center for Morphometric Analysis. 2016.
17. Bates D, Mächler M, Bolker B, Walker S. Fitting Linear Mixed-Effects Models Using lme4. Journal of Statistical Software. 2015; 67(1), 1–48. doi: 10.18637/jss.v067.i01

© 2025 Cantoni V et al. *JAMA Network Open*.

18. Grassi M, Palluzzi F, Tarantino B. SEMgraph: an R package for causal network inference of high-throughput data with structural equation models Bioinformatics. 2022; 38(20):4829-4830. doi: 10.1093/bioinformatics/btac567.

© 2025 Cantoni V et al. *JAMA Network Open*.
