# Supplementary material for: Home-Based Gamma Transcranial Alternating Current Stimulation in Patients With Alzheimer Disease: A Randomized Clinical Trial
Source: JAMA Netw Open. 2025 Dec 8;8(12):e2546556. doi: 10.1001/jamanetworkopen.2025.46556 (PMC12687098; doi:10.1001/jamanetworkopen.2025.46556)
Supplement: Supplement 3. — Data Sharing Statement [file jamanetwopen-e2546556-s003.pdf]

Data Sharing Statement

Cantoni. Home-Based Gamma Transcranial Alternating Current Stimulation in Patients With Alzheimer Disease. *JAMA Netw Open*. Published December 08, 2025.
doi:10.1001/jamanetworkopen.2025.46556

Data
Additional Information: ClinicalTrials.gov (NCT05643326)
https://clinicaltrials.gov/study/NCT05643326?term=borroni%20&rank=4
Data available: No
